# AdipoRon promotes angiogenesis in B-cell leukemia by modulating pro-angiogenic factors through AdipoR1

**DOI:** 10.1007/s11033-025-11183-x

**Published:** 2025-11-11

**Authors:** Marta Mallardo, Rosita Russo, Aurora Daniele, Angela Chambery, Ersilia Nigro

**Affiliations:** 1 Department of Molecular and Biotechnological Medicine, University of Naples “Federico II”, Naples, 80138 Italy; 2 CEINGE-Biotechnologies Advances S.c.a r.l., Via G. Salvatore 486, Naples, 80145 Italy; 3 Dipartimento di Scienze e Tecnologie Ambientali, Università della Campania “Luigi Vanvitelli”, Via Vivaldi 43, Biologiche, Farmaceutiche, Caserta, 81100 Italy

**Keywords:** AdipoRon, JVM-2 cells, Leukemia, AdipoRs, Angiogenesis, VEGF-A

## Abstract

**Background:**

Adiponectin, a cytokine predominantly secreted by adipose tissue, is increasingly recognized for its role in cancer, including hematologic malignancies. However, its involvement in leukemia-related angiogenesis remains poorly understood. Here, we investigated the effects of AdipoRon, an adiponectin agonist, on angiogenesis in the JVM-2 lymphoblastic cell line, a model for B-cell leukemia.

**Methods:**

The effects of AdipoRon were assessed on human umbilical vein endothelial cells (HUVECs) and JVM-2 cells using the tube formation assay. The expression of VEGF receptors, VEGF-A, HIF-1α, and CXCL-1 were investigated at both mRNA by quantitative real-time PCR and protein levels by ELISA and Western Blotting. The role of AdipoR1 and AdipoR2 in mediating AdipoRon effects was investigated by siRNA-mediated silencing of each receptor.

**Results:**

AdipoRon significantly promoted angiogenesis in HUVECs, both directly and through the secretion of soluble factors from JVM-2 cells. Treatment with AdipoRon upregulated VEGF-A and its receptors, with VEGF-R2 showing the most prominent increase. Additionally, both HIF-1α and CXCL-1 were up-regulated following AdipoRon administration. Finally, silencing of AdipoR1, but not AdipoR2, diminishes the AdipoRon-induced upregulation of the angiogenesis-related factors, suggesting that AdipoR1 is the primary receptor mediating the effects of adiponectin in leukemia cells.

**Conclusion:**

Our findings demonstrated that AdipoRon promotes angiogenesis in B-cell leukemia by enhancing pro-angiogenic factors through AdipoR1highlighting adiponectin’s significance in angiogenesis. A better understanding of adiponectin’s mechanisms could facilitate the development of therapeutic strategies targeting its pathway to inhibit tumor angiogenesis, offering promising approaches for leukemia treatment. Further studies are needed to fully explore adiponectin potential in leukemia.

**Graphical abstract:**

**Figure:**
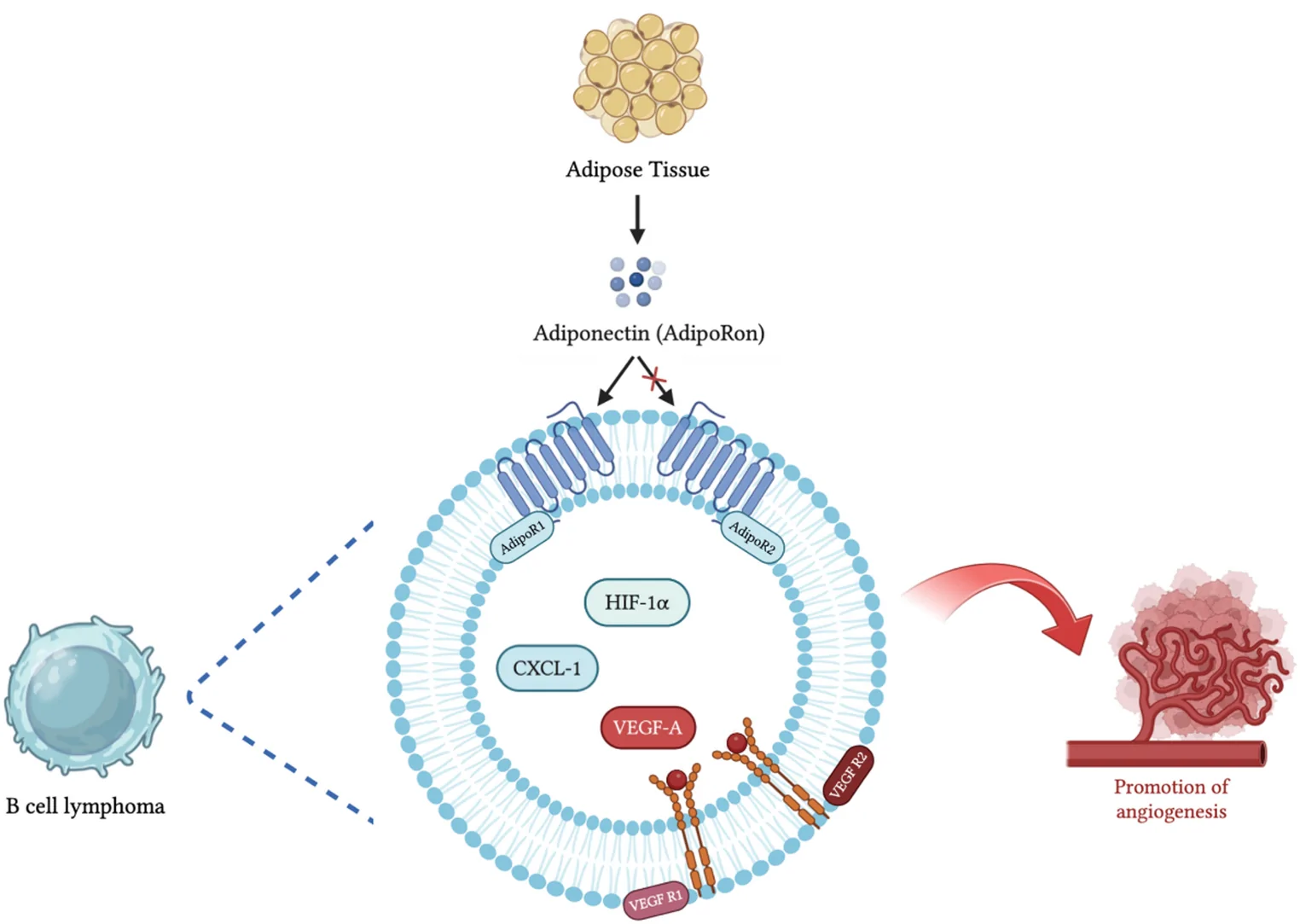

**Supplementary Information:**

The online version contains supplementary material available at https://doi.org/10.1007/s11033-025-11183-x.

## Introduction

Adiponectin, a cytokine predominantly secreted by adipose tissue, plays a pivotal role in metabolic regulation, inflammation, and vascular homeostasis [1]. Its insulin-sensitizing, anti-inflammatory, anti-atherogenic, and anti-proliferative properties have garnered significant attention in oncology, positioning adiponectin as a potential modulator of tumor progression [2].

In solid tumors, circulating adiponectin levels are generally reduced compared to healthy individuals, with lower concentrations often correlating with increased tumor aggressiveness and poor prognosis [3]. This inverse relationship has been observed across various malignancies, including breast, colorectal, and endometrial cancers, suggesting a protective role of adiponectin in solid tumor development [4–6].

Conversely, in hematologic malignancies, the role of adiponectin remains more complex, with conflicting evidence reported [7]. Indeed, circulating adiponectin levels have been shown to either increase, remain unchanged, or even decrease [8–11]. Retrospective studies have reported hyperadiponectinemia in patients with both adult and childhood non-Hodgkin lymphoma (NHL), as well as Hodgkin lymphoma (HL), compared to control groups, with higher adiponectin levels being associated with poorer prognosis and worse clinical outcomes [8, 10, 12]. On the other hand, smaller studies involving acute lymphoblastic leukemia (ALL) patients have observed lower adiponectin levels [9, 13]. Interestingly, a separate retrospective study found no significant differences in adiponectin levels when comparing ALL survivors to healthy controls [14]. Therefore, the involvement of adiponectin in B-cell leukemia remain poorly understood.

Angiogenesis is a critical process not only in solid tumors but also in hematologic malignancies, supporting leukemic cell survival, proliferation, and dissemination [15, 16]. Key regulators of angiogenesis include vascular endothelial growth factor A (VEGF-A), which promotes endothelial cell proliferation and migration; hypoxia-inducible factor 1-alpha (HIF-1α), orchestrating cellular responses to hypoxia and upregulating pro-angiogenic factors like VEGF-A; and chemokine (C-X-C motif) ligand 1 (CXCL1), stimulating endothelial cell migration and proliferation [17–19].

Adipose tissue, and in particular adiponectin, has been increasingly implicated in angiogenic events over the past decade [20–22]. Our group has recently shown that adiponectin can influence angiogenic responses in endothelial cells by increasing the expression of VEGF-A, CXCL1, matrix metalloproteinase-2 (MMP-2), and 9 (MMP-9) [23]. While adiponectin has been extensively studied in the context of solid tumors, its role in leukemia-associated angiogenesis remains largely unexplored.

Given the lack of data on the role of adiponectin in angiogenesis within lymphoblastic cells, the aim of the present study was to investigate the in vitro effects of AdipoRon, a small-molecule agonist of endogenous adiponectin, on angiogenesis in the JVM-2 lymphoblastic cell line, which serves as a model for B-cell leukemia. Next, we focused on the impact of AdipoRon on the secretome of JVM-2 cells, highlighting the secretion of molecules among which VEGF-A. The expression of additional factors implicated in angiogenesis were also investigated (HIF-1α, and CXCL1). Finally, we aimed to determine whether AdipoRon’s effects on angiogenesis in leukemia are mediated by AdipoR1 and/or AdipoR2.

## Materials and methods

### Cell culture

The human lymphoblast cell line (JVM-2) was kindly provided by the Bank of Human and Animal Continuous Cell Lines-CEINGE Biotecnologie Avanzate “Franco Salvatore”, Napoli, Italy. Cells were cultured in Rosewell Park Memorial Institute 1640 Medium (RPMI 1640) (Thermo Fisher Scientific, Massachusetts, USA), supplemented with 10% fetal bovine serum (Lonza, Basel, Switzerland), 1% L-glutamine (Sigma-Aldrich, MO, USA), 1% penicillin/streptomycin (Thermo Fisher Scientific, Massachusetts, USA). The cells were grown in a 5% CO2 humidified incubator, at 37 °C. All experiments were conducted in RPMI medium containing different concentrations of AdipoRon (5.5, 11.5, and 23 µM). The selected AdipoRon doses were based on previously published evidence and adapted to the type of experiment according to the biological response analyzed. This explains the use of different concentrations across experiments [24].

### Generation of JVM-2 conditioned medium

To obtain conditioned medium (CM) from JVM-2 cells, we treated the cells for 48 h with increasing concentrations of AdipoRon (5.5, 11.5, and 23 µM). After treatment, the cell medium was collected by centrifugation at 13,000 rpm for 10 min and then stored at − 80 °C until use.

### HUVEC tube formation assay

The ability of HUVEC endothelial cells to form capillary-like structures (tubes) following AdipoRon administration and exposure to JVM-2 conditioned medium (CM) was assessed using the in vitro Gibco^®^ Angiogenesis Starter Kit (Thermo Fisher Scientific, Massachusetts, USA). The CM used in this assay was collected from JVM-2 cells after 48 h of treatment with AdipoRon (11.5, 23 µM), centrifuged at 13.000 rpm for 10 min, and stored at − 80 °C until use. The cells were seeded into coated 24-well plates and treated with AdipoRon (11.5, 23 µM) and CM for 4 h. Cells incubated in medium alone served as the negative control, while those treated with VEGF-A (10 ng/mL) (Applied Biological Materials Inc., BC, Canada) were used as the positive control. Network-like structures were examined under an inverted microscope (objective, ×4). Tube-like structures were defined as endothelial cord formations connected at both ends. The number of branching points was quantified by counting five random fields per well under a microscope, as previously reported [23].

### RNA extraction and quantitative real time-PCR

Total RNA was extracted from JVM-2 cells by using TRIzol Reagent (Thermo Fisher Scientific, Massachusetts, USA). The RNA concentration was quantified by using fluorescence-based detection with Qubit 4 Fluorometer (Thermo Fisher Scientific, Massachusetts, USA). One microgram of total RNA was subjected to reverse transcription with SuperScript IV VILO Master Mix (Invitrogen, Massachusetts, USA) according to the manufacturer’s instructions. Gene expression analysis was carried out using the C1000 Touch Thermal Cycler (Bio-Rad, CA, USA) and PowerUp SYBR Green Master Mix (Applied Biosystems, Massachusetts, USA). GAPDH was used as housekeeping gene; fold changes were calculated with the 2^−ΔΔCt^ method as previously reported [25]. Primer sequences used for q-RT-PCR are available upon request. The experiments were conducted twice in duplicate.

### Targeted quantitative analysis of secreted cytokines by Bio-Plex assay

The targeted quantitative analysis of secreted cytokines and chemokines in samples was performed using the Bio-Plex multiplex system (Bio-Rad, Milan, Italy) based on xMAP technology [26]. Conditioned media for Bio-plex determinations were collected from the same number of cells (5 × 10^5^ cells) for both AdipoRon-treated (23 µM for 48 h) JVM-2 and untreated cells. Normalization of analyte concentrations to cell numbers was not required, since fold change ratios were not affected by differences in cell number for the two conditions under investigation. Samples were prepared and analyzed in triplicate. Magnetic beads labeled with red and infrared fluorophores, are coated with specific antibodies, thus allowing the simultaneous detection of multiple target analytes within one sample. Following reaction of beads with target analytes, detection is performed with a biotinylated antibody and phycoerythrin conjugated streptavidin. All steps were performed according to manufacturer’s instructions. The concentration of the following analytes were detected simultaneously: interleukin (IL)−1β, IL-1ra, IL-2, IL-4, IL-5, IL-6, IL-7, IL-8, IL-9, IL-10, IL-12 (p70), IL-13, IL-15, IL-17 A, IP10, eotaxin, granulocyte-colony stimulating factor (G-CSF), Granulocyte macrophage colony stimulating factor (GM-CSF), Interferon (IFN)γ, monocyte chemoattractant protein 1 (MCAF/MCP-1), Macrophage inflammatory protein 1-alpha and beta (MIP-1α and MIP-1β), RANTES, tumour necrosis factor alpha (TNF-α), platelet-derived growth factor-BB (PDGF-BB), Vascular endothelial growth factor (VEGF), Basic fibroblast growth factor (FGF-basic). Each sample was tested in triplicate. Data were acquired using a Bio-Plex MAGPIX Multiplex Reader system (Bio-Rad). Standard curves optimization and the calculation of analyte concentrations were performed by using the Bio-Plex Manager software. Cytokines concentration was expressed in pg/mL.

### ELISA measurement of VEGF-A and CXCL1 secreted into the culture medium

The release of VEGF-A and CXCL1 from JVM2 cells into the culture media was quantified using the human VEGF-A and CXCL1 ELISA kits (Invitrogen, BMS277-2 and BMS2122, Massachusetts, USA) following the manufacturer’s protocol. The media used for this assay were collected from JVM-2 after 48 h of treatment with AdipoRon (5.5, 11.5, and 23 µM), The experiments were performed twice in duplicate, as previously described [23].

### Western blotting

Total protein content was extracted from cells using pre-cooled radioimmunoprecipitation assay (RIPA) buffer (Sigma-Aldrich, MO, USA) containing protease inhibitor cocktail (Abcam, Cambridge, UK). Later, protein content was quantified by the Bradford’s method (Bio-Rad, CA, USA). Successively, samples were diluted in Laemmli buffer 4X and boiled for 5 min at 95 °C.Thirty µg of total cellular protein were loaded onto a polyacrylamide gel and separated by SDS-PAGE, transferred to PVDF membranes (Pierce Biotechnology, Massachusetts, USA) and blocked with 5% non-fat milk. The membranes were then incubated overnight at 4 °C with the following primary antibodies, according to the manufacturer’s instructions: HIF-1α and GAPDH (Cell Signaling Technology, Massachusetts, USA), AdipoR1 (Santa Cruz Biotechnology, Texas, USA), and AdipoR2 (Phoenix Pharmaceuticals Inc., CA, USA). The next day, the membranes were incubated with anti-rabbit and anti-goat antibodies conjugated to horseradish peroxidase (Cell Signaling Technology, Massachusetts, USA). Finally, protein bands were detected using the ChemiDoc XRS system (Bio-Rad, CA, USA) with ECL detection reagents (Pierce Biotechnology, Massachusetts, USA).

### SiRNAs mediated transfection of AdipoR1 and AdipoR2

JVM-2 cells were cultured in their standard growth medium in a 6-well plate. Once the cells reached approximately 80% confluence, they were transfected with 50 nM siRNA targeting AdipoR1 (Ambion Inc., CA, USA) and 100 nM siRNA targeting AdipoR2 (Dharmacon, Colorado, USA), or with non-silencing control siRNA (Ambion Inc., CA, USA) using Lipofectamine™ 3000 (Invitrogen, Massachusetts, USA), following the manufacturer’s instructions. After 24 h of siRNA transfection, cells were collected, and gene silencing efficiency was assessed by qPCR and Western blot. (supplementary Fig. 1).

The siRNA-transfected cells were then treated for an additional 48 h with AdipoRon (5.5, 11.5, and 23 µM).

### Statistical analysis

Data are expressed as mean ± standard deviation (SD) of replicates. Comparisons between control and treated groups were conducted using two-tailed Student’s t-test, or one-way or two-way ANOVA followed by Tukey’s multiple comparisons test. A p-value < 0.05 was considered statistically significant. Expression levels of secreted cytokineswere expressed as mean ± SD. Student’s t-test (two-tailed) was employed to assess the significance between the CM from AdipoRon-treated cells with respect to control samples by using the GraphPad Prism software v 8.4.2 (La Jolla, CA, USA). Statistical significance thresholds were defined as following: (*) p-values ≤ 0.05, (**) p-values ≤ 0.01, (***) p-values ≤ 0.001.

## Results

### Adiporon administration enhances HUVEC angiogenesis

**Fig. 1:**
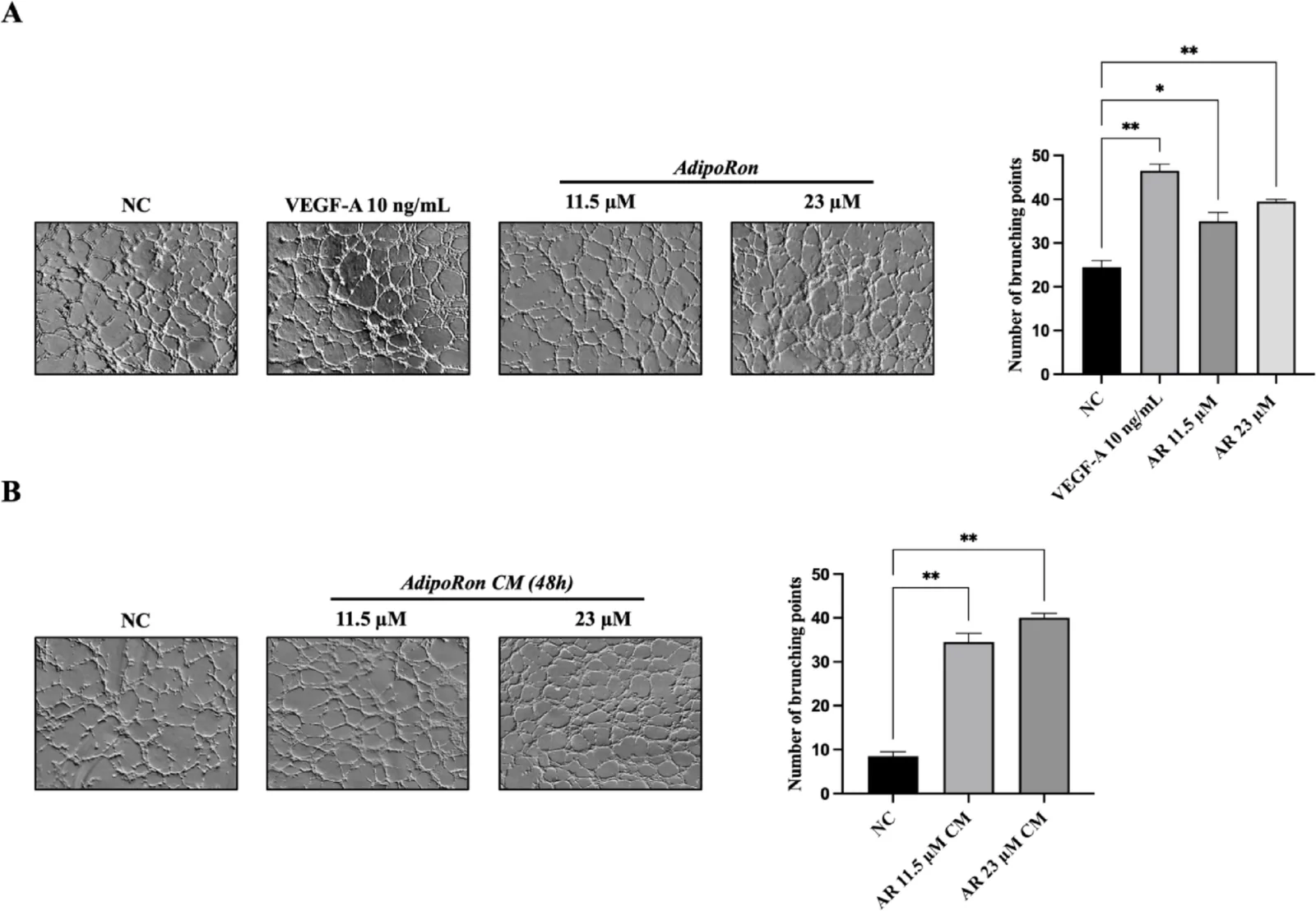
Angiogenesis in HUVEC cells is stimulated by AdipoRon treatment and CM collected from JVM-2. HUVEC endothelial cells were treated with AdipoRon (11.5, 23 µM) and conditioned media (CM) derived from JVM-2 cells treated with AdipoRon (11.5, 23 µM) for 4 h. (**A**) Representative images of tube formation of HUVEC cells treated with AdipoRon (left) and their quantization (right). (**B**) Representative images of tube formation of HUVEC cells treated with CM derived from JVM-2 cells treated with AdipoRon (left) and their quantization (right). Untreated cells were considered as the negative control (NC); cells treated with VEGF-A (10 ng/mL) were considered as a positive control. Data are reported as mean ± SD of two independent experiments performed in duplicate. ∗*p* < 0.05; ∗∗*p* < 0.01 versus NC

To directly evaluate the effect of AdipoRon on angiogenesis, we performed an in vitro HUVEC tube formation assay. HUVEC endothelial cells were treated for 4 h with AdipoRon (11.5, 23 µM) or CM derived from JVM-2 cells treated with AdipoRon (11.5, 23 µM for 48 h) (see Fig. 1). Both concentrations of AdipoRon significantly promoted angiogenesis compared to untreated cells (Fig. 1, panel A) in a similar extent to that of VEFG. Notably, as shown in Fig. 1, panel B, CM from JVM-2 cells also significantly enhanced tube formation. These findings suggest that AdipoRon induces angiogenesis in HUVEC cells both directly and indirectly, supporting the hypothesis that the angiogenic response may, at least in part, be mediated by the secretion of soluble factors.

### Secretome profiling of cytokine and chemokine cell signaling molecules

To investigate the secreted factors that are modulated by AdipoRon treatment, we compared the expression levels of secreted cytokines and chemokines in CM of JVM-2 cells treated with AdipoRon (23 µM for 48 h) with respect to CM from untreated cells, using a multiplexed immunoassay-based platform. We observed slight changes in the expression levels of eotaxin, IL-2, IFN-γ, and TNF-α (supplementary Fig. 1). Notably, VEGF was significantly upregulated in the CM from AdipoRon-treated cells compared to controls, strongly supporting the pro-angiogenic effects observed. Given that VEGF showed the most prominent increase among the analyzed factors, we selected it for further investigation to better understand its contribution to AdipoRon-induced angiogenesis.

### VEGF receptors are expressed in JVM-2 cells, with their mRNA levels rising in response to adiporon administration

**Fig. 2:**
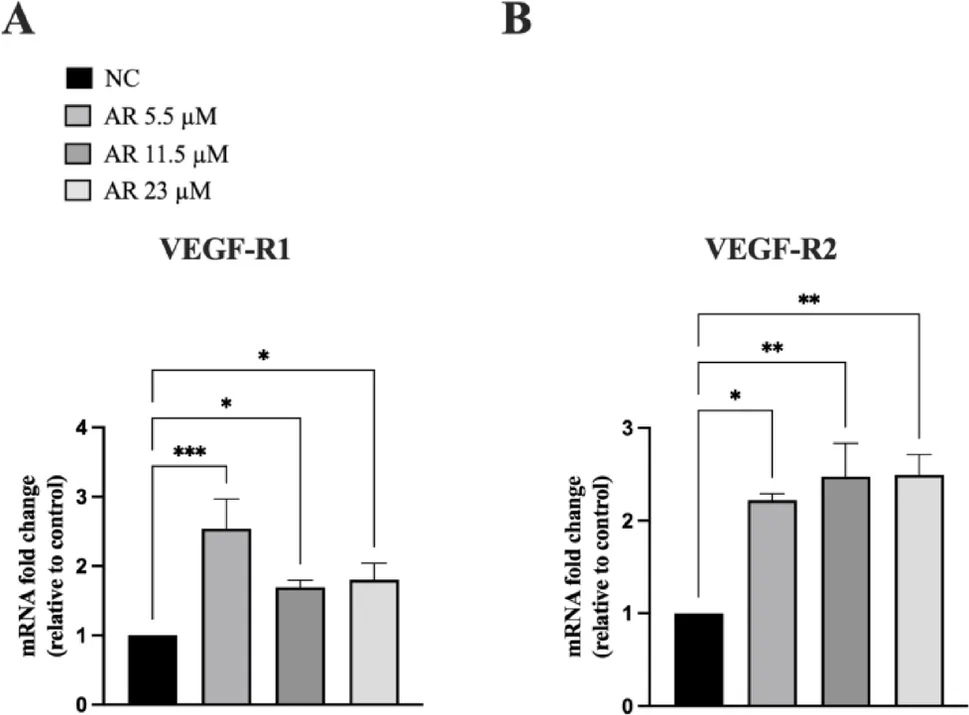
VEGF-A receptors are expressed in JVM-2 cells and up-regulated by AdipoRon. JVM-2 cells were treated with AdipoRon (5.5, 11.5, and 23 µM) for 48 h and the expression of VEGF-A receptors was evaluated. (**A**, **B**) VEGF-A type 1 (**A**) and 2 (**B**) receptors mRNA expression was quantified by qPCR using GAPDH as endogenous control. All the data are shown as mean ± SD of two independent experiments performed in duplicate. ∗*p* < 0.05; ∗∗*p* < 0.01 and ∗∗∗*p* < 0.001 versus NC

Based on the above findings and given the pivotal role of VEGF-A as a key regulator of the angiogenic process, we analyzed the expression of VEGF-A receptors in JVM-2 cells. We confirmed the presence of both VEGFRs at the mRNA level (see Fig. 2). Next, JVM-2 cells were treated with AdipoRon (5.5, 11.5, and 23 µM) for 48 h, and the mRNA expression levels of VEGF R1 and VEGF R2 were assessed *via* qPCR. AdipoRon treatment significantly increased the mRNA expression of both VEGF receptors, with VEGF R2 showing a stronger upregulation at all tested doses (Fig. 2, panels A and B).

### Adiporon treatment promotes the angiogenesis in JVM-2 cells by upregulating VEGF-A, HIF-1α, and CXCL-1

**Fig. 3:**
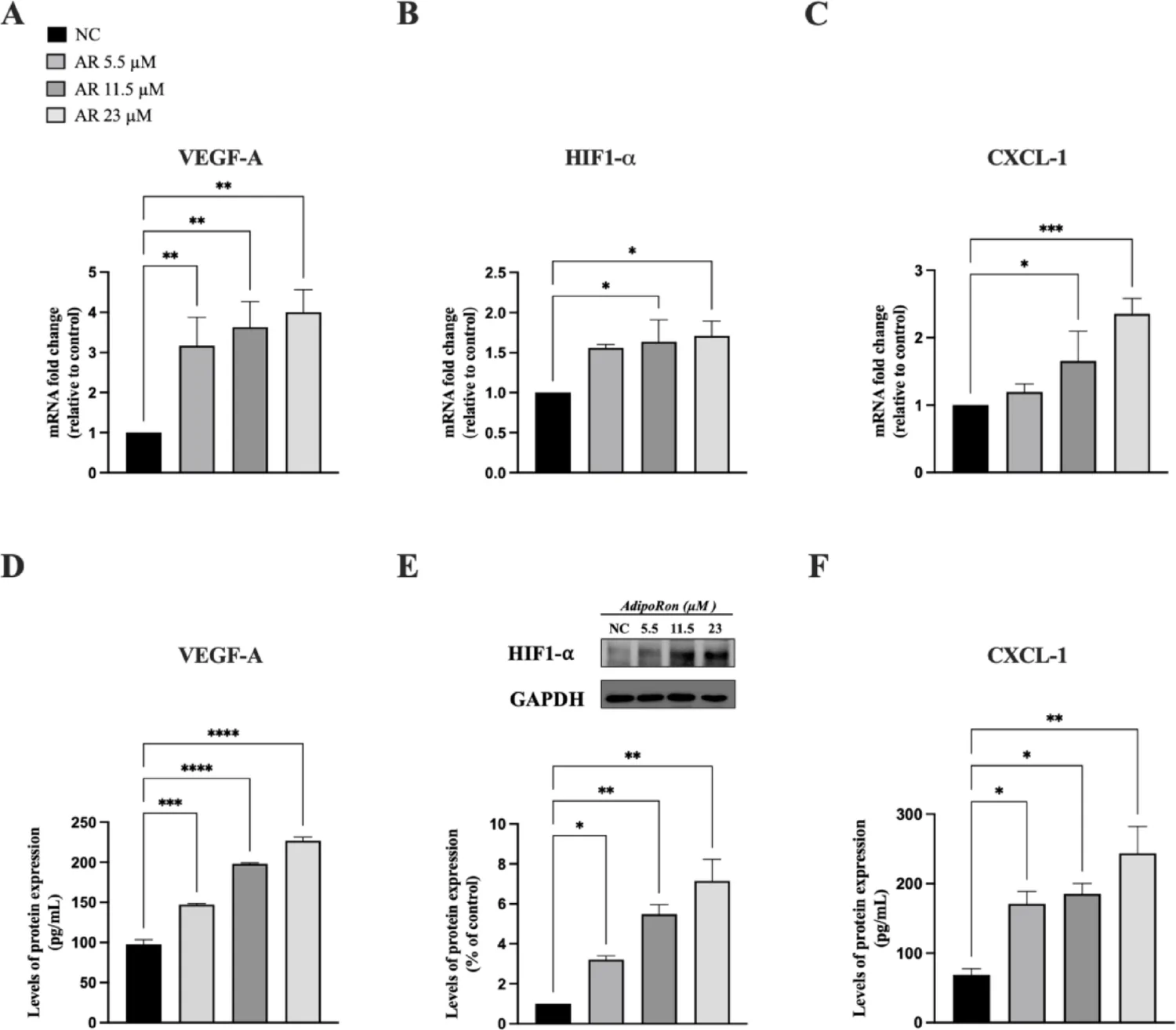
AdipoRon induces the expression of VEGF-A, HIF-1α, and CXCL-1 at both mRNA and protein levels. JVM-2 cells were treated with AdipoRon (5.5, 11.5, and 23 µM) for 48 h and the expression of VEGF-A, HIF-1α, and CXCL-1was evaluated. (**A**, **D**) VEGF-A mRNA expression was determined by q-PCR (**A**) and the data were standardized using GAPDH and subsequently quantified via the 2^−ΔΔCt^ method while its release in the culture medium was determined by ELISA (**D**). (**B**,** E**) HIF-1α mRNA expression was evaluated by qPCR (**B**), whereas HIF-1α protein levels were analyzed by western blotting (E), using GAPDH as an internal loading control. (C, F) CXCL-1 mRNA levels were analyzed by q‐PCR (**C**) using GAPDH as endogenous control and its release in the culture medium was determined by ELISA (**F**). Untreated cells were used as negative control (NC). All the data are shown as mean ± SD of two independent experiments performed in duplicate. ∗*p* < 0.05; ∗∗*p* < 0.01; ∗∗∗*p* < 0.001 and ∗∗∗∗*p* < 0.0001 versus NC

To assess the potential effects of AdipoRon on angiogenesis-related pathways, we treated cells with increasing concentrations of AdipoRon (5.5, 11.5, and 23 µM) for 48 h and analyzed both mRNA and protein levels of the angiogenesis-related factors VEGF-A, HIF-1α, and CXCL-1 (see Fig. 3).

Our results demonstrated a significant increase in VEGF-A mRNA levels following AdipoRon administration, accompanied by a corresponding rise in its protein concentration in the extracellular medium (Fig. 3, panels A and D). Additionally, we evaluated the effects of AdipoRon on two additional key angiogenesis-related proteins, HIF-1α and CXCL-1. As shown in Fig. 3, panels B and E, AdipoRon significantly increased HIF-1α expression at the mRNA level and enhanced its secretion into the extracellular medium. Similarly, CXCL-1 expression was upregulated at both the transcriptional and protein levels (Fig. 3, panels C and F).

Overall, these findings suggest that AdipoRon promotes angiogenesis in JVM-2 cells by upregulating VEGF-A, HIF-1α, and CXCL-1, with VEGF R2 likely being the primary receptor involved.

### Silencing of AdipoR1 but not AdipoR2 reduces the angiogenesis in JVM-2 cells, by downregulating VEGF-A, CXCL-1, and HIF-1α

To determine the receptor subtype(s) responsible for AdipoRon effects on VEGF-A, HIF-1α, CXCL-1 and VEGF receptors levels, the expression of AdipoR1 or AdipoR2 was selectively inhibited using specific siRNAs. JVM-2 cells were transiently transfected with siRNA targeting AdipoR1 (50 nM) or AdipoR2 (100 nM) or with a non-silencing control siRNA for 24 h, followed by treatment with AdipoRon (5.5, 11.5, and 23 µM) for 48 h.

**Fig. 4:**
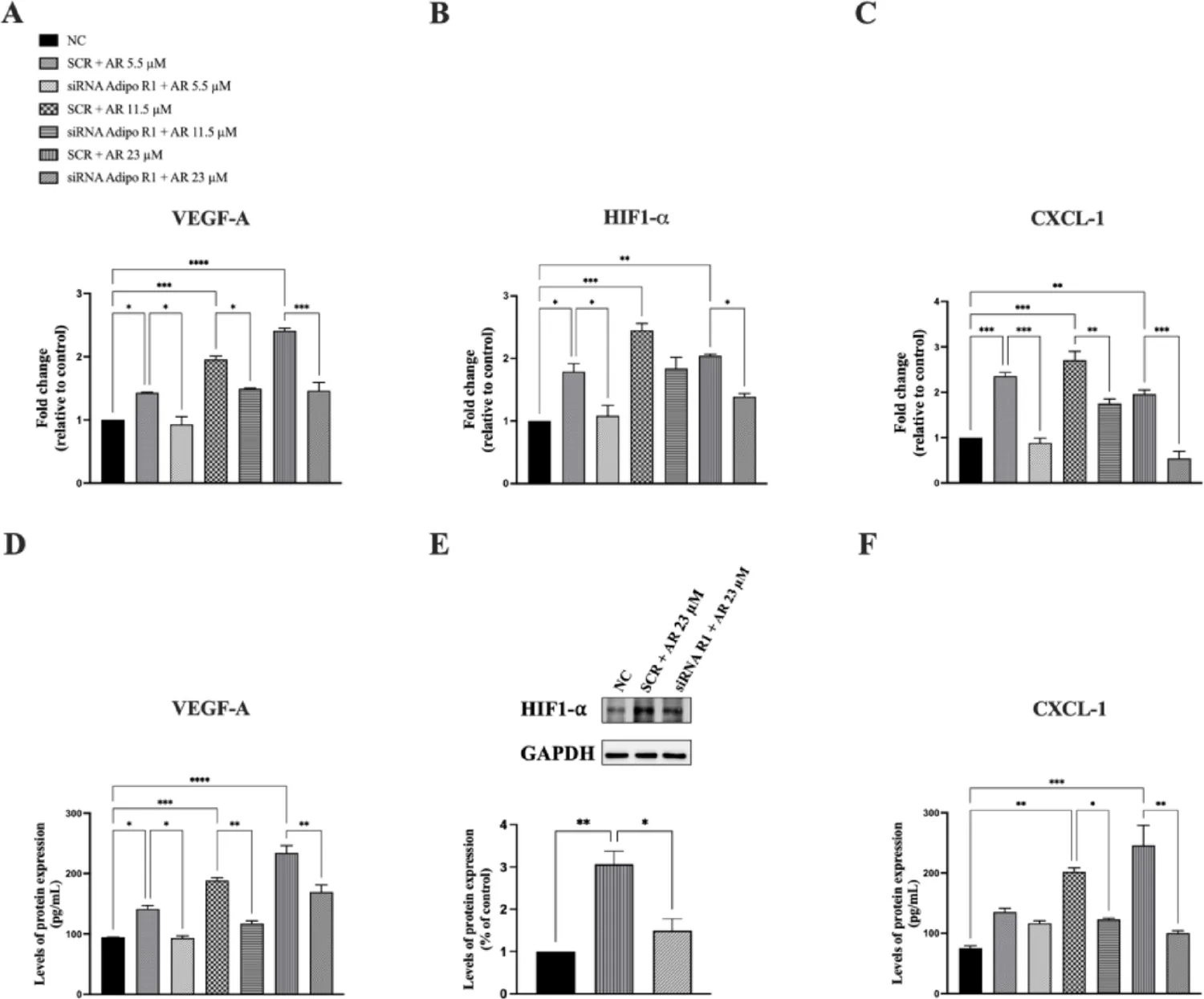
AdipoR1 silencing significantly suppresses AdipoRon-mediated increase of the expression of VEGF-A, HIF-1α and CXCL-1 at both mRNA and protein levels. JVM-2 cells were transiently transfected with siRNA targeting AdipoR1 (50 nM) or with a non-silencing control siRNA for 24 h, followed by treatment with AdipoRon (5.5, 11.5, and 23 µM) for 48 h and the effects on VEGF-A, CXCL-1 and HIF-1α expression were evaluated. (**A**, **D**) VEGF-A mRNA expression was assessed by qPCR (A) and the data were normalized to GAPDH and quantified using the 2^−ΔΔCt^ method while its levels in the culture medium were measured by ELISA (D). (**B**, **E**) HIF-1α mRNA expression was evaluated by qPCR (**B**), whereas HIF-1α protein levels were analyzed by western blotting (**E**), using GAPDH as an internal loading control. (**C**, **F**) CXCL-1 mRNA expression was assessed by qPCR (C), with GAPDH as the endogenous control and its levels in the culture medium were measured by ELISA (**F**). Untreated cells served as the negative control (NC). Data are presented as mean ± SD from two independent experiments performed in duplicate. *p < 0.05; **p < 0.01; ***p < 0.001 versus NC

**Fig. 5:**
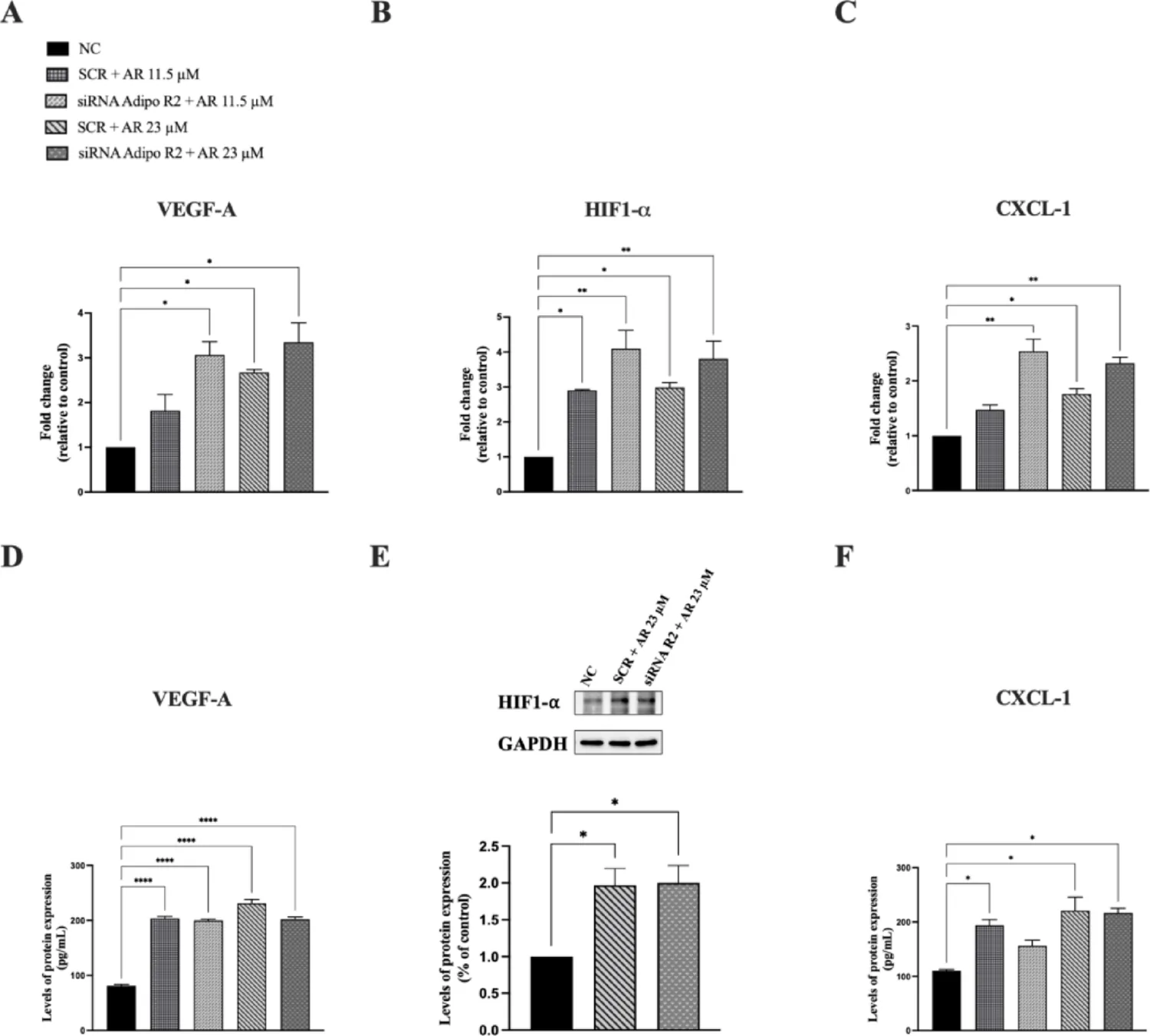
AdipoR2 silencing does not affect AdipoRon-induced pro-angiogenic signaling in JVM-2 cells. JVM-2 cells were transiently transfected with siRNA targeting AdipoR2 (100 nM) or with a non-silencing control siRNA for 24 h. Following transfection, cells were treated with AdipoRon at concentrations of 5.5, 11.5, and 23 µM for 48 h, and the impact on VEGF-A, CXCL-1 and HIF-1α expression was evaluated. (**A**, **D**) VEGF-A mRNA expression was quantified by qPCR (**A**), with data normalized to GAPDH and calculated using the 2 − ΔΔCt method levels while its levels in the culture medium were measured by ELISA (**D**). (**B**, **E**) HIF-1α mRNA expression was determined by qPCR (**B**), while HIF-1α protein levels were analyzed by western blotting (**E**), using GAPDH as an internal loading control. (**C**, **F**) CXCL-1 mRNA expression was assessed by qPCR (**C**), with GAPDH as the endogenous control and its levels in the culture medium were measured by ELISA (**F**). Untreated cells were used as the negative control (NC). The data are expressed as mean ± SD from two independent experiments performed in duplicate. *p < 0.05; **p < 0.01; ***p < 0.001 compared to NC

AdipoR1 silencing significantly reduced AdipoRon-induced VEGF-A expression at both the mRNA and protein levels (Fig. 4, panels A and D). Additionally, AdipoR1 silencing strongly suppressed AdipoRon-induced HIF-1α mRNA and protein expression (Fig. 4, panels B and E), and a similar reduction was observed for CXCL-1 (Fig. 4, panels C and F). Conversely, AdipoR2 silencing did not significantly impact the AdipoRon-mediated increase in the expression of the aforementioned pro-angiogenic factors (Fig. 5).

**Fig. 6:**
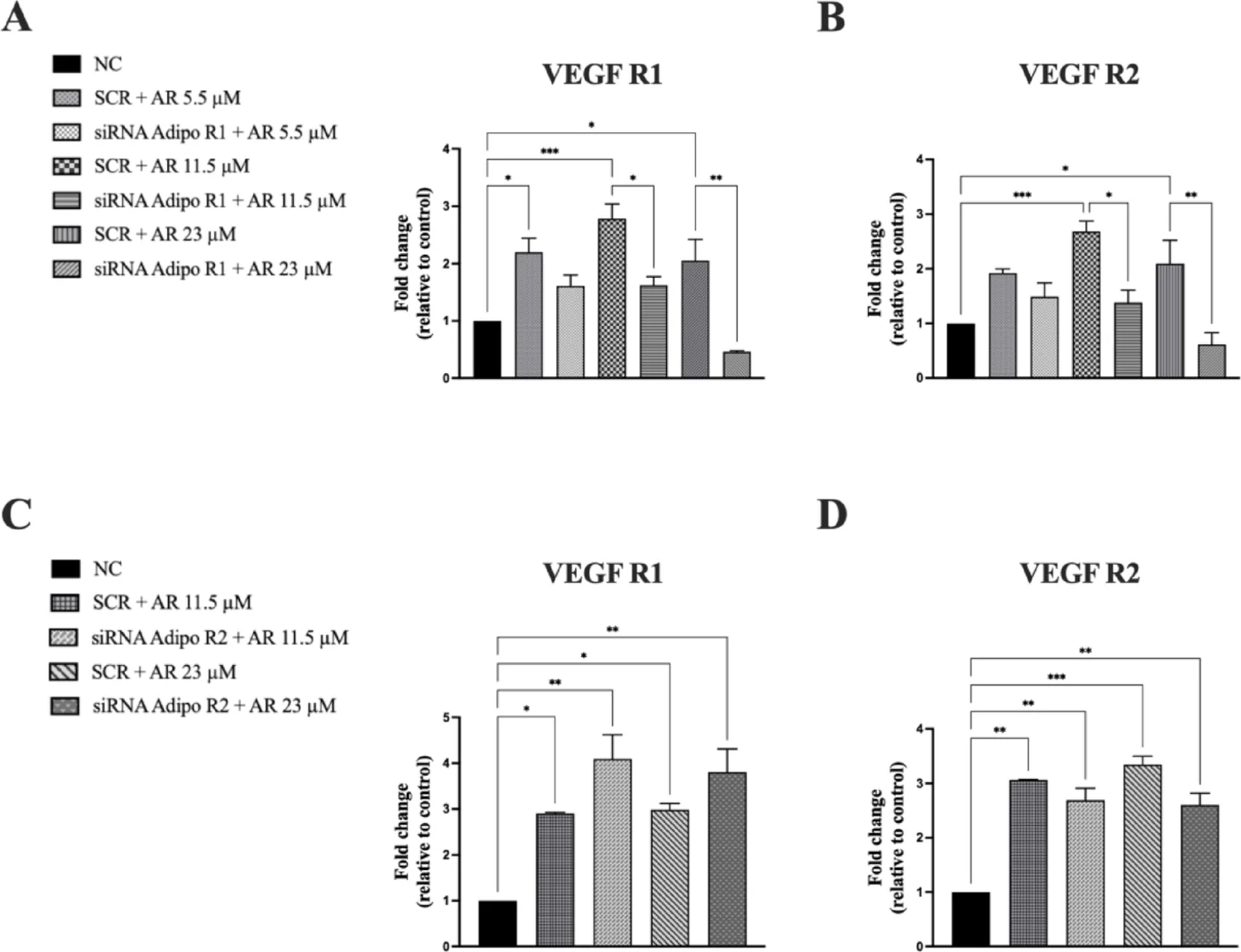
AdipoR1 silencing significantly suppresses AdipoRon-mediated increase of the expression of VEGF R1 and VEGF R2, whereases AdipoR2 silencing does not affect AdipoRon induced effect. JVM-2 cells were transiently transfected with siRNA targeting AdipoR1 (50 nM) or AdipoR2 (100 nM) or with a non-silencing control siRNA for 24–48 h, followed by treatment with AdipoRon (5.5, 11.5, and 23 µM) for 48 h and the effects on VEGF receptors 1 and 2 expression were evaluated. (**A**, **C**) mRNA expression of VEGF receptors 1 was assessed by qPCR after silencing of AdipoR1 (**A**) and Adipo R2 (**C**), with GAPDH serving as the endogenous control. (**B**, **D**) mRNA expression of VEGF receptors 2 was assessed by qPCR after silencing of AdipoR1 (**B**) and Adipo R2 (**D**), with GAPDH serving as the endogenous control. *p < 0.05; **p < 0.01; ***p < 0.001 compared to NC

In fact, after AdipoR2 inhibition, both their mRNA and protein levels remained comparable to those observed in AdipoRon-treated cells, suggesting that AdipoR2 does not play a major role in mediating the pro-angiogenic effects of AdipoRon in JVM-2 cells. Interestingly, the stimulatory effects of AdipoRon on VEGF receptor mRNA levels were also significantly reduced by AdipoR1 inhibition (Fig. 6, panels A and B). and not by AdipoR2 silencing (Fig. 6, panels C and D). These findings highlight the selective involvement of AdipoR1 in regulating the angiogenic signaling pathways induced by AdipoRon.

## Discussion

Adiponectin, a cytokine abundantly secreted by adipose tissue, has been widely studied for its role in several physiological and pathological processes, including metabolic regulation, inflammation, and cancer [1]. However, its involvement in angiogenesis, particularly in hematologic malignancies, remains poorly understood [13, 20, 27, 28]. Therefore, we investigated the effects of AdipoRon (an adiponectin agonist) on the angiogenesis process in relation to leukemia cancer: our data support a role of AdipoRon in the induction of endothelial angiogenesis through the positive regulation of VEGF-A, HIF-1α and CXCL-1 in JVM-2 cells, a model of B-cell leukemia. These findings are in line with our previous data showing a pro-growth effect of adiponectin toward JVM-2 cells [24]. We also assessed which adiponectin receptor was primarily involved in mediating the angiogenic response: our data provide compelling evidence that AdipoR1, but not AdipoR2, is responsible for the signaling underlying the pro-angiogenic phenotype in B-cell leukemic cells.

In recent years, adiponectin has emerged as a key player in oncology research, as numerous studies have highlighted its dysregulation in various malignancies, particularly those associated with obesity [29–33]. In the context of blood malignancies, circulating adiponectin levels have been found to be either increased or decreased in different types of leukemia [13, 34, 35]. Epidemiological studies have suggested a potential association between low adiponectin concentrations and hematologic malignancies, including non-Hodgkin’s lymphoma (NHL), although findings remain controversial [10, 12, 36].

Pathological angiogenesis has been recognized as a critical event not only in the growth of solid tumors but also in various hematological malignancies [36]. In fact, although research in the latter area remains limited, growing evidence suggests the presence of dysregulated angiogenesis in some leukemia subtypes [38, 39]. In this context, the endocrine function of adipose tissue in modulating angiogenesis represents a promising area of research [22]. Among adipokines, in vitro studies have shown that adiponectin could play a role in endothelial cell proliferation and new vessel formation in relation to cancer, although contradictory studies have suggested that adiponectin could suppress or promote angiogenesis [20, 23, 40–42].

Here, we demonstrated that AdipoRon promotes a pro-angiogenic phenotype in JVM-2 cells, as evidenced by the induction of tube formation in HUVECs. Analysis of the secretome from AdipoRon-treated JVM-2 cells revealed a modest increase in the expression of eotaxin, IL-2, IFN-γ, and TNF-α, along with a more pronounced upregulation of VEGF, suggesting its pivotal role in mediating AdipoRon’s pro-angiogenic effects. In addition to VEGF-A, other key angiogenic factors such as HIF-1α and CXCL-1 were also elevated following AdipoRon treatment. These findings align with previous studies indicating that adiponectin signaling can stimulate VEGF-A-dependent angiogenesis [21, 42]. Furthermore, [42] showed that adiponectin promotes angiogenesis in response to ischemic stress in endothelial cells; its absence impaired angiogenic repair, while its supplementation improved tissue perfusion, highlighting the crucial role of adiponectin in supporting vascular regeneration and maintaining angiogenic capacity [42]. Similarly, Ouchi et al. (2004) demonstrated that adiponectin enhances angiogenesis via activation of AMPK and Akt signaling pathways in endothelial cells [39]. Conversely, [43] reported anti-angiogenic effects of adiponectin in a mouse model of laser-induced choroidal neovascularization, where it suppressed VEGF expression in choroidal tissue [43]. Mahadev et al. [44] also observed that adiponectin inhibited VEGF-induced migration of human coronary endothelial cells, supporting its potential role as a negative regulator of angiogenesis under certain conditions [44]. Importantly, we observed a strong upregulation of VEGFR2 at all tested doses of AdipoRon, indicating that this receptor plays a pivotal role in mediating the angiogenic effects observed. This is particularly relevant, as accordingly to previous research, VEGFR2 is the primary receptor responsible for transmitting VEGF-induced pro-angiogenic signals, leading to endothelial cell proliferation and vascular remodeling [45].

In our cell model, alongside VEGF-A upregulation, we also observed an increased expression of HIF-1α and CXCL-1, two proteins closely associated with the angiogenic process. Both are well-known regulators of endothelial recruitment and neovascularization, and their secretion by tumor cells has been linked to enhanced angiogenesis and immune cell infiltration in several cancer models [42, 47]. The increased levels of HIF-1α and CXCL1 observed in our study further support the pro-angiogenic potential of AdipoRon. To our knowledge, there is no evidence on the effects of adiponectin on HIF-1α and CXCL1 expression specifically in JVM-2 cells. However, studies in other cancer cell models have shown that adiponectin can enhance CXCL1 secretion, which in turn promotes VEGF release and angiogenesis [40, 42]. Moreover, it has been reported that preconditioning mesenchymal stem cells with AdipoRon enhances cell survival, migration, and angiogenesis by increasing the expression of HIF-1α, C-X-C chemokine receptor type 4 (CXCR4), C-C chemokine receptor type 2 (CCR2), VEGF, MMP-2, and MMP-9 [47]. These findings are consistent with our results, reinforcing the hypothesis that AdipoRon-induced upregulation of VEGF-A, HIF-1α, and CXCL-1 may contribute to the establishment of a pro-angiogenic microenvironment, depending on the tumor context.

It is to notice that that both direct AdipoRon treatment and exposure to AdipoRon-conditioned medium significantly enhanced endothelial cell tube formation, reinforcing the notion that JVM-2 cells may release soluble angiogenic factors in response to AdipoRon stimulation. This is consistent with our previous study, which demonstrated that AdipoRon enhances the angiogenic capacity of HUVEC cells [23]. Consistent with our findings, Ouchi et al. demonstrated that adiponectin acts as a chemoattractant, promoting HUVEC endothelial cell migration and stimulating their differentiation into capillary-like structures [39]. However, contrasting evidence suggest that adiponectin may exert inhibitory effects on angiogenesis in certain contexts. Bråkenhielm et al. demonstrated that adiponectin suppresses endothelial cell proliferation and migration in vitro and remarkably prevents new blood vessel growth in vivo [48].

A different involvement of both adiponectin receptors in hematologic malignancies has been reported [49]. Thus, a crucial objective of our study was to determine which adiponectin receptor subtype mediates the observed angiogenic effects. Our data revealed that silencing AdipoR1, but not AdipoR2, significantly suppressed the AdipoRon-induced increase in these angiogenic factors, indicating that AdipoR1 is the primary receptor responsible for mediating AdipoRon’s angiogenic signals in JVM-2 cells. Specifically, AdipoR1 inhibition led to a marked reduction in VEGF-A and VEGFR2 expression, strongly suggesting that AdipoRon might promote angiogenesis through the AdipoR1/VEGF-A/VEGFR2 axis. This finding aligns with previous studies showing that AdipoR1 plays a dominant role in metabolic and angiogenic signaling pathways, whereas AdipoR2 is more involved in lipid metabolism and oxidative stress regulation [50]. The lack of a significant effect following AdipoR2 silencing further confirms that AdipoRon primarily acts *via* AdipoR1 to regulate angiogenesis in leukemia cells.

There are some limitations to this study. First, we used an immortalized tumor cell line as a disease model instead of primary lymphoblasts, which may not fully capture the complexity of leukemia in patients. Future studies should include additional cell models to validate these findings. Second, this study lacks in vivo validation. Since all experiments were conducted in vitro, they do not account for the complex interactions within the body that influence angiogenesis. Further research using animal models are necessary to confirm whether AdipoRon has similar effects in a physiological environment. Lastly, the molecular signaling mechanisms underlying AdipoRon’s effects in JVM-2 cells remain incompletely understood. Given that adiponectin can activate multiple downstream pathways, further studies are needed to clarify which specific signaling cascades drive the observed pro-angiogenic effects.

## Conclusion

In conclusion, our findings underscore the role of adiponectin, particularly through its receptor AdipoR1, in promoting angiogenesis in B-cell leukemia. Treatment with AdipoRon enhanced angiogenic activity in endothelial cells, likely through the upregulation of key pro-angiogenic factors such as VEGF-A, HIF-1α, and CXCL-1. These results suggest that adiponectin contributes to tumor vascularization and may represent a potential novel therapeutic target.

A more comprehensive understanding of the molecular mechanisms underlying adiponectin signaling could pave the way for innovative therapeutic strategies aimed at modulating tumor angiogenesis. Targeting the adiponectin pathway may offer new opportunities for the treatment of leukemia. Nevertheless, further investigations, particularly in vivo, are essential to validate these findings and to fully explore the clinical potential of adiponectin-based interventions in cancer treatment.

## Supplementary Information

Below is the link to the electronic supplementary material.

Supplementary Material 1

Supplementary material 2 (DOCX 13.0 kb)

## Data Availability

No datasets were generated or analysed during the current study.
