# Supplementary figures and images for: AdipoRon promotes angiogenesis in B-cell leukemia by modulating pro-angiogenic factors through AdipoR1
Source: Mol Biol Rep. 2025 Nov 11;53(1):67. doi: 10.1007/s11033-025-11183-x (PMC12605524; doi:10.1007/s11033-025-11183-x)

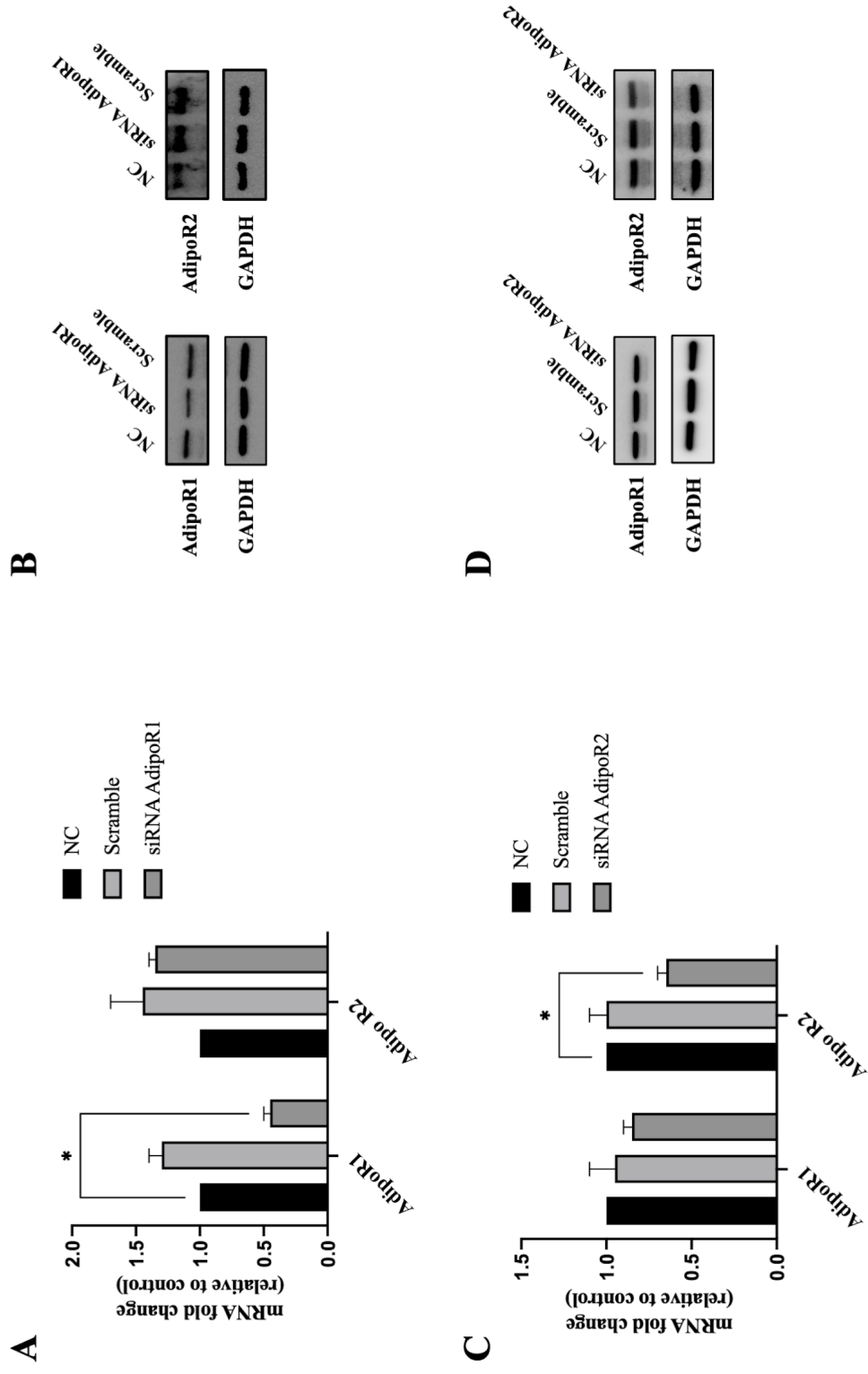

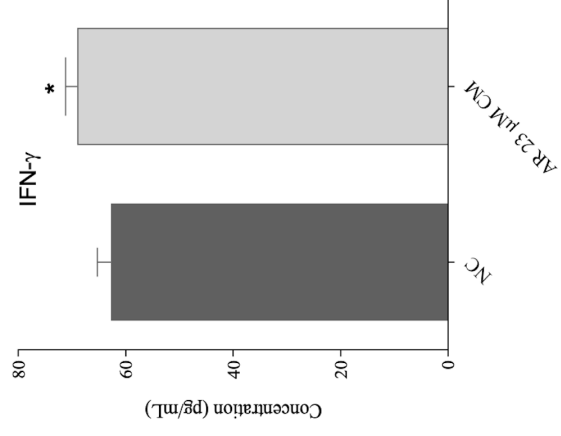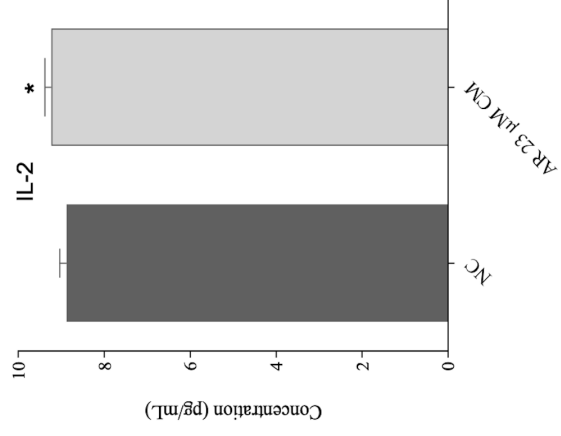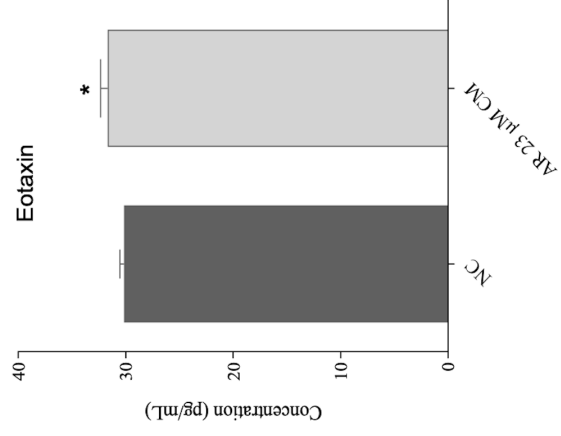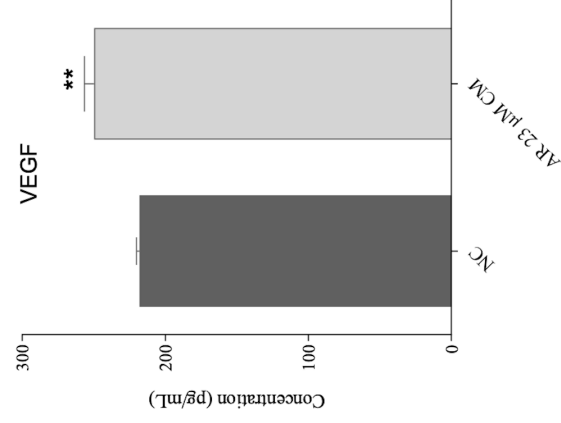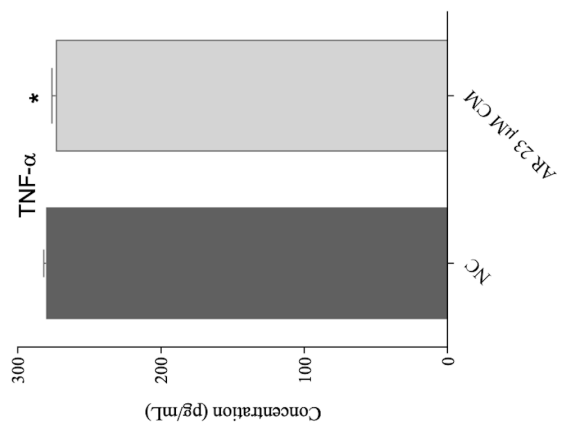

Supplement: Supplementary file 1 — Supplementary Material 1 [file 11033_2025_11183_MOESM1_ESM.pdf]
