# Supplementary material for: AdipoRon promotes angiogenesis in B-cell leukemia by modulating pro-angiogenic factors through AdipoR1
Source: Mol Biol Rep. 2025 Nov 11;53(1):67. doi: 10.1007/s11033-025-11183-x (PMC12605524; doi:10.1007/s11033-025-11183-x)
Supplement: Supplementary file 2 — Supplementary material 2 (DOCX 13.0 kb) [file 11033_2025_11183_MOESM2_ESM.docx]

**Supplementary materials**

**Silencing of adiponectin receptors AdipoR1 and AdipoR2 *via* siRNA transfection**

To investigate the contribution of adiponectin signaling to the effects of AdipoRon on JVM-2 cells, specific siRNAs targeting both AdipoRs were used, and transfection efficiency was assessed by both qPCR and western blotting. Regarding AdipoR1, compared to the control siRNA, which had no effect on AdipoR1 expression, AdipoR1-specific siRNA induced a 55% inhibition at the mRNA level, without affecting AdipoR2 expression (Figure 1, panel A). Reduced AdipoR1 protein expression was confirmed by western blotting (Figure 1, panel B).

Regarding AdipoR2, compared to the control siRNA, which had no impact on AdipoR2 expression, AdipoR2-specific siRNA induced a 35% reduction at the mRNA level, without affecting AdipoR1 expression. The decrease in AdipoR2 expression was further validated by western blotting.

**Supplementary Figure 1. qPCR and western blotting validation of the transfection efficiency of AdipoR1 and AdipoR2 siRNAs.** JVM-2 cells were transfected with siRNA for AdipoR1 and AdipoR2 for 24 h together with control siRNA. The effects of siRNA were verified both with q-PCR and western blotting. (A, C) mRNA levels of AdipoR1 and AdipoR2 after transfection of siRNA targeting AdipoR1 (A) and siRNA targeting AdipoR2 (C). (B, D) Representative Western blot showing AdipoR1 and AdipoR2 expression after transfection with AdipoR1-specific siRNA (B) and AdipoR2-specific siRNA (D). Values are given as mean ± SD of two experiments performed in duplicate. * *p* < 0.05 versus NC.

**Supplementary Figure 2.** Expression levels of cytokines and chemokines detected in CM from AdipoRon-treated JVM-2 cells (JVM TRT) with respect to control (JVM CTR). Measured concentrations are referred to CM collected from 5 × 10^5^ cells for both AdipoRon-treated JVM-2 and untreated cells. CM were simultaneously screened for determining the cytokines concentration by interpolation on properly generated standard curves. All measurements were performed in triplicate. Data are reported as means ± SD. *p < 0.05; **p < 0.01; ***p < 0.001 AdipoRon-treated JVM-2 versus JVM-2 control dataset.
